# State-wise variation and inequalities in caesarean delivery rates in India: analysis of the National Family Health Survey-5 (2019–2021) data

**DOI:** 10.1016/j.lansea.2024.100512

**Published:** 2024-12-03

**Authors:** Rohini Dutta, Priyansh Nathani, Priti Patil, Rakhi Ghoshal, Shagun Tuli, Juul M. Bakker, Alex J. van Duinen, Nobhojit Roy, Adeline A. Boatin, Anita Gadgil

**Affiliations:** a Program in Global Surgery and Social Change, Department of Global Health and Social Medicine, Harvard Medical School, Boston, MA, USA; b Mary Horigan Connors Center for Women’s Health and Gender Biology, Brigham and Women’s Hospital, Boston, MA, USA; c Hinduhridaysamrat Balasaheb Thackeray Medical College and Dr Rustom Narsi Cooper Municipal General Hospital, Mumbai, India; d Department of Statistics, BARC Hospital, Mumbai, India; e Gender Justice, CARE, USA; f Department of Obstetrics and Gynecology, Hurley Medical Center, MI, USA; g Department of Public Health and Nursing, Norwegian University of Science and Technology (NTNU), Trondheim, Norway; h Department of Surgery, St. Olav’s Hospital, Trondheim University Hospital, Trondheim, Norway; i Department of Global Public Health, Karolinska Institutet, Sweden; j The George Institute for Global Health, New Delhi, India; k Department of Obstetrics and Gynecology, Massachusetts General Hospital, Boston, MA, USA

**Keywords:** Cesarean section delivery, India, NFHS-5, Private sector, Inequality

## Abstract

**Background:**

India’s caesarean delivery (CD) rate of 21.5% suggests adequate national access to CD but may mask significant disparities. We examined variation in CD rates across states (geography), wealth, and health care sector (public versus private). We also aimed to determine relative inequality in CD rates across wealth quintiles.

**Methods:**

The current study was a cross-sectional analysis of CD rates from the National Family Health Survey-5 (2019–2021) disaggregated by asset-based household wealth quintiles for each state and by healthcare sector (public versus private). Data from 724,115 women aged 15–49 years across 28 states and eight union territories were analysed. Women who reported their most recent live birth within the past five years were included. Relative inequality was measured by comparing CD rates in the richest versus the poorest quintiles.

**Findings:**

Disaggregating the national CD rate of 21.5% showed substantial variation in CD rate across states, ranging from 5.2% in Nagaland to 60.7% in Telangana and across wealth quintiles, ranging from 0% to 76.7% (Assam). CD facility rates were higher in private than public facilities across all wealth quintiles. Over two-thirds of states (69%) had at least twice the CD rate in the richest wealth quintile versus the poorest quintile. Relative inequality in CD rates between the richest and poorest was 5.3 nationally and was higher in public (4.0) versus private (1.4) facilities.

**Interpretation:**

The national CD rate in India masks complex geographical, wealth, and sector-related inequalities in CD utilisation. Accounting for these variations is imperative when interpreting national-level rates to better assess the equity in the distribution of CD services.

**Funding:**

None.

**Research in context**

**Evidence before this study**

Previous research in India has primarily focused on analysing national level and overall caesarean delivery (CD) rates. However, it is well known that the attainment of a seemingly ‘optimal’ CD rate at the national level does not necessarily equate to equitable or adequate access, for marginalised and disadvantaged communities. Evidence suggests that wealth status, among other factors, is crucial in determining an individual’s access to CD. There is a notable gap in understanding the access disparity between public and private healthcare facilities across India, with the wealth index as an explanatory variable conspicuously absent.

**Added value of this study**

This work was based on the largest nationally representative sample of Indian women who have undergone CD. This study disaggregated the national CD rate, identifying disparities in access to CD service provision based on geography, household income, and type of healthcare facilities (public and private). This study also demonstrated that wealth disparities in CD utilisation also occur within private facilities, with fewer CDs in the least wealthy and more CDs in the wealthiest populations. The study concluded that both people with high-income and people with low-income in India receive more CDs in private than public facilities.

**Implications of all the available evidence**

This study highlights the importance of disaggregating CD rates to examine the effectiveness of government policies in their aim to provide access to CD for the economically disadvantaged and marginalised communities in India.

## Introduction

Caesarean delivery (CD) is the most commonly performed surgical procedure globally and is vital to safeguard the lives of both mothers and neonates.^1^^,^^2^ Globally, 21% of women give birth by CD, and projections suggest that this figure is expected to increase to 28% by 2030.^3^^,^^4^ However, the effectiveness of increasing CD rates in reducing maternal and perinatal mortality is uncertain, as the CD rates vary significantly by region. At the population level, a CD rate above 10–19% has not been linked to a decrease in maternal and perinatal mortality rates.^2^ Increasing population-level rates above 19% thus raise concerns regarding equitable access to safe, timely, and affordable CD for all those who require it.^3^, ^4^, ^5^

Inequalities can be defined as the observed differences in CD rates between different population subgroups,^6^ measuring the normative concept of inequities in CD access. The WHO emphasises ensuring equitable access to CD for all women in need of the procedure over achieving the previously considered ‘optimal’ population-level CD rate of 10–15%.^7^ Disparities in CD rates indicate unequal access to the procedure among different groups and reflect a dual scenario; low rates indicate that women requiring the procedure may not have adequate access, resulting in maternal and newborn mortality and morbidity.^8^ On the other hand, high rates are suggestive of overuse without medical necessity, which is associated with higher rates of adverse outcomes (infection, haemorrhage, surgical complications) and misallocation of resources.^9^ Studies have revealed that seemingly acceptable national-level rates may conceal underlying within-country inequities, highlighting the need for a more detailed analysis.^10^^,^^11^ Disaggregating CD rates can identify vulnerable groups that might lack access to CD.^10^^,^^12^^,^^13^ Globally, a positive correlation has been observed between CD access and financial capacity,^14^ with wealth quintiles serving as a yardstick for household economic status.^11^ Understanding the impact of economic status on CD rates is crucial for developing interventions that address inequities across and within countries.^10^^,^^15^^,^^16^ This approach also directs attention to social factors that might influence local and regional rates, aiding policymakers in improving outcomes for disadvantaged populations.^10^^,^^12^^,^^13^

In India, CD rates have steadily increased. Data from the National Family Health Surveys (NFHS) demonstrate an increase in the CD rates 8.5% in 2005–2006, 17.2% in 2015–2016, and 21.5% in 2019–2021.^17^^,^^18^ Despite government schemes introducing subsidised CD in public hospitals in India, access to economically disadvantaged populations remains limited.^3^^,^^19^^,^^20^ For example, *Janani Suraksha Yojana* (JSY) is a government scheme that provides cash assistance for institutional deliveries, including CDs, to women living below the poverty line.^19^ The nationwide reach of the JSY stands at 36.4% of the target population, with significant variations observed across regions and socioeconomic groups.^21^

High CD rates in private hospitals have largely contributed to the rising rates of CD in India. Data from 2019 to 2021 show that 21.4% of all institutional deliveries and 47.5% of CDs are performed in private facilities.^18^ Previous research has explored factors impacting CD rates, including demographic and socio-cultural variables. However, limited evidence exists on the correlation between state-level CD rates and the population’s economic status.^22^^,^^23^ The primary objective of this study was to analyse variations in CD rates across India, both overall and when stratified by wealth quintiles and healthcare sectors to quantify inequalities.

## Methods

### Data source

We performed a secondary analysis on the National Family Health Survey-5 (NFHS-5) conducted during 2019–2021. The NFHS in India is a comprehensive and large-scale cross-sectional household survey. The International Institute for Population Sciences (IIPS) implements it under the direction of the Ministry of Health and Family Welfare. The NFHS is modelled on the Demographic Health Survey (DHS) and aims to provide information on population, health, and family welfare for India.^18^^,^^24^ This includes data on maternal and child health, family planning, fertility, nutrition, and other related aspects. Collected at both state and district levels, the data serves as a valuable resource for policymakers, implementers, and administrators, aiding them in crafting evidence-based strategies. The most recent iteration, the fifth round, comprises representative samples from urban and rural households across all 28 states and 8 Union Territories (UTs).^18^ India currently has eight union territories including Andaman and Nicobar Islands, Chandigarh, Dadra and Nagar Haveli and Daman and Diu, Delhi, Jammu and Kashmir, Ladakh, Lakshadweep, and Puducherry.^25^

### Data collection

Field work for the NFHS-5 was conducted in two phases: Phase-I from June 17, 2019 to Jan 30, 2020 and Phase-II from Jan 2, 2020 to April 30, 2021. The process of data collection involved 1061 field teams. The survey coordinators from each Field Agency determined the allocation of ‘primary sampling units’ to the teams. Interviewers were mandated to make a minimum of three callbacks if a participant was unavailable during the household interview or the initial visit. Four separate questionnaires (household, woman’s, man’s, and biomarker) were administered in 18 local languages through Computer-Assisted Personal Interviewing.^26^ The content of the survey questionnaire was approved by the review board of IIPS and ICF, USA. The disseminated survey had data quality assurance and quality control mechanisms.^27^

The woman’s questionnaire encompassed a wide variety of health parameters such as state-level institutional birth and CD in private and government facilities, antenatal care, delivery care, family planning services, and demographic information including literacy rates.^18^

### Study variables

Our primary outcome variable was caesarean delivery. The question “*Was the baby delivered by caesarean section, that is, did they cut your belly open to take the baby out*?” was asked to eligible women (defined as women of reproductive age, 15–49 years, who reported a live birth within the five years preceding the survey). Women who responded “*yes*” were categorised as having undergone a “delivery by caesarean section”. CD rates were calculated by dividing the number of CDs by the total number of live births and stillbirths for each state or UT, expressed as a percentage. In this paper, we used population-level CD rates from the NFHS-5 survey. These rates encompass a specific population within a geographic area. CD rates based on facility type are derived from self-reported household survey responses, not directly from healthcare facilities, offering insights into delivery practices.

The wealth quintiles and health facility type (public or private) were considered explanatory variables. The wealth index serves as an indicator of the economic status of households and is presented in the survey datasets as a background characteristic. While the survey does not directly collect data on consumption or income, it does gather detailed information on household characteristics, as well as access to a range of consumer goods, services, and assets like ownership of television, refrigerators, housing conditions, and other related factors. The wealth index is constructed using household asset data as proxies for long-term wealth via principal components analysis.^28^ This pre-calculated index was used to categorize the five wealth quintiles (poorest, poorer, middle, richer, and richest) for all states and UTs to capture the relative economic positioning of households. Detailed wealth index calculation is mentioned in the official NFHS documentation.^29^

### Data analysis

The NFHS dataset includes variables related to wealth quintiles and CD summarised by state and UT. A cross-tabulation was created to present the distribution of CD rates across different wealth quintiles. The CD rates were calculated for all wealth quintile groups for each state and UT. We calculated relative inequality in CD rates across states and UTs by dividing the rate in the richest by that in the poorest wealth quintile.

We assessed the statistical significance by calculating 95% CIs for relative inequality measures. Relative measures were deemed insignificant if the 95% CI included one. We considered wealth quintiles for states or UTs with CD rates less than 10% as underuse and rates of more than 10% were considered as overuse. This cut-off point was based on its previous usage in literature, and is only indicative, not prescriptive. There is no consensus on the ideal rate or range for CDs.^8^^,^^12^^,^^13^ The 10% threshold was drawn from studies showing improved maternal and neonatal outcomes at or above this rate, and was selected for this study as bare minimum rate needed especially in regions with limited surgical care.^12^ We also analysed the proportions of CD rates by health facility type (public and private). The relative inequalities between CD rates in public and private facilities were assessed separately for each wealth quintile.

Box plots were prepared to illustrate the distribution of CD rates among various wealth quintiles and between public and private healthcare facilities. We used these tools to directly observe and compare the variability and central tendencies of CD rates within each group. By employing box plots, we identified the median, range, and any outliers in the CD rates across different economic levels and types of healthcare facilities to reveal any disparities and trends in CD practices. Statistical analysis and graphical representation were performed using SPSS version 22.0 (SPSS Inc., Chicago, Illinois, USA) for Windows, RStudio and Microsoft Excel® (Microsoft, Redmond, Washington, USA), and the study followed STROBE guidelines.

### Ethical approval

This study is based on the anonymised data available in the public domain from the NFHS-5 survey. The local ethics committee of the IIPS determined that formal ethics approval was not needed to use this data for research.^30^

### Role of the funding source

There was no funding source for this study.

## Results

Data were available for 724,115 women aged 15–49 years, representing 636,699 households with a response rate of 88–95%. Of this sample, 93.9% of women who had a live birth in the five years preceding the survey registered the pregnancy as their most recent live birth.

### National variation in caesarean delivery rates between states

**Fig. 1:**
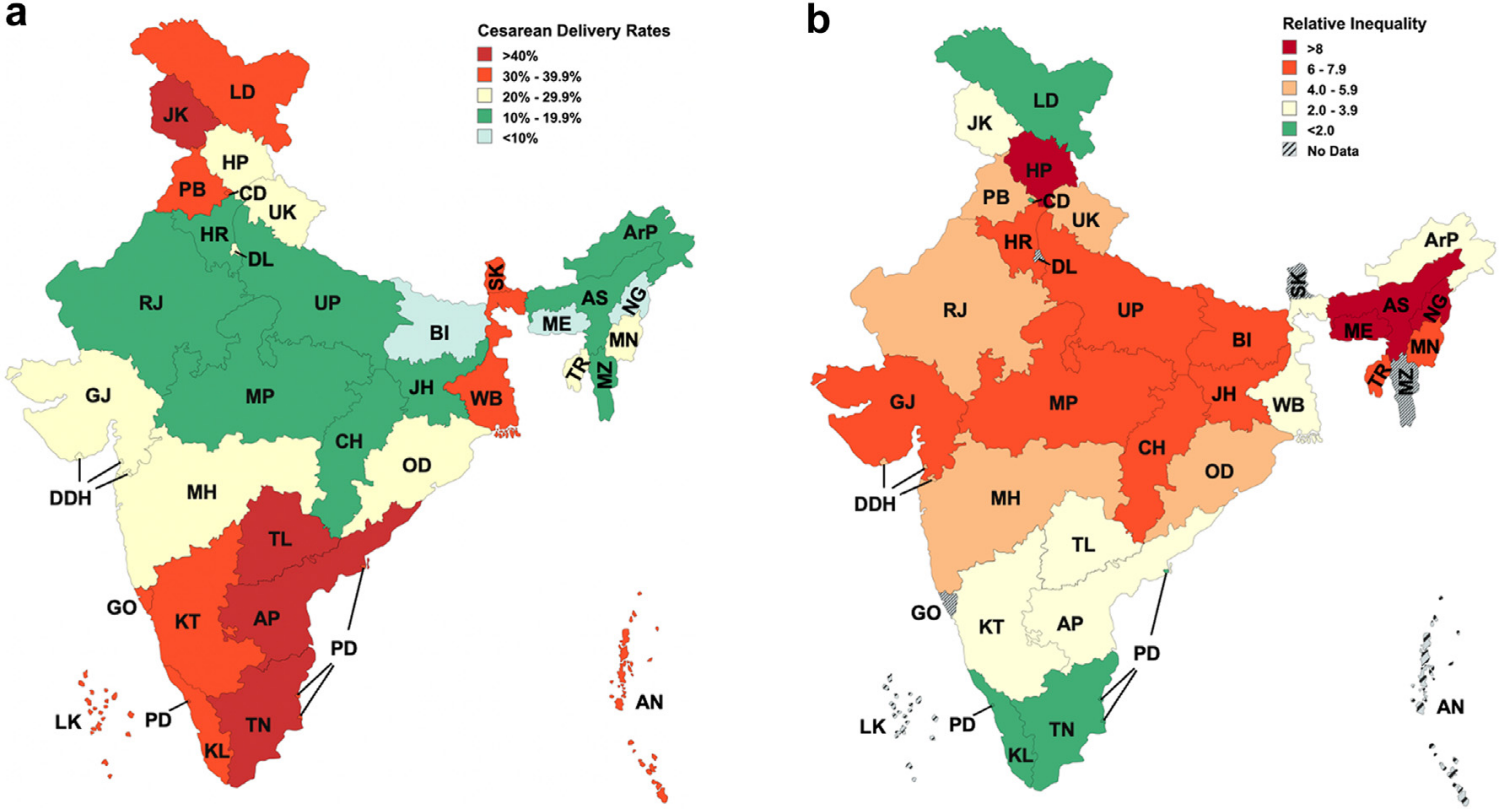
**a) Caesarean delivery rates in India b) Relative inequalities in caesarean delivery rates in India.** AP: Andhra Pradesh, ArP: Arunachal Pradesh, AS: Assam, BI: Bihar, CH: Chhattisgarh, GJ: Gujarat, GO: Goa, HP: Himachal Pradesh, HR: Haryana, Jh: Jharkhand, KL: Kerala, KT: Karnataka, ME: Meghalaya, MH: Maharashtra, MN: Manipur, MP: Madhya Pradesh, MZ: Mizoram, NG: Nagaland, OD: Odisha, PB: Punjab, RJ: Rajasthan, SK: Sikkim, TL: Telangana, TN: Tamil Nadu, TR: Tripura, UK: Uttarakhand, UP: Uttar Pradesh, WB: West Bengal, AN: Andaman & Nicobar Islands, CD: Chandigarh, DDH: Dadra Nagar Haveli & Diu Daman, DL: Delhi, JK: Jammu & Kashmir, LD: Ladakh, LK: Lakshadweep, PD: Puducherry.

The national CD rate is 21.5% (CI: 21.3%–21.7%), ranging from 5.2% in Nagaland to 60.7% in Telangana. The southern states of Telangana (60.7%, CI: 59.4%–62%), Tamil Nadu (44.9%, CI: 44%–45.9%), and Andhra Pradesh (42.4%, CI: 41.3%–43.6%) had the highest CD rates, whereas, the eastern states of Nagaland (5.2%, CI: 2.2%–8.1%), Meghalaya (8.2%, CI: 6.4%–10%), and Bihar (9.7%, CI: 9.4%–10%) had the lowest CD rates (Supplementary Table S1, Fig. 1a). Caesarean delivery rates by economic status.

**Fig. 2:**
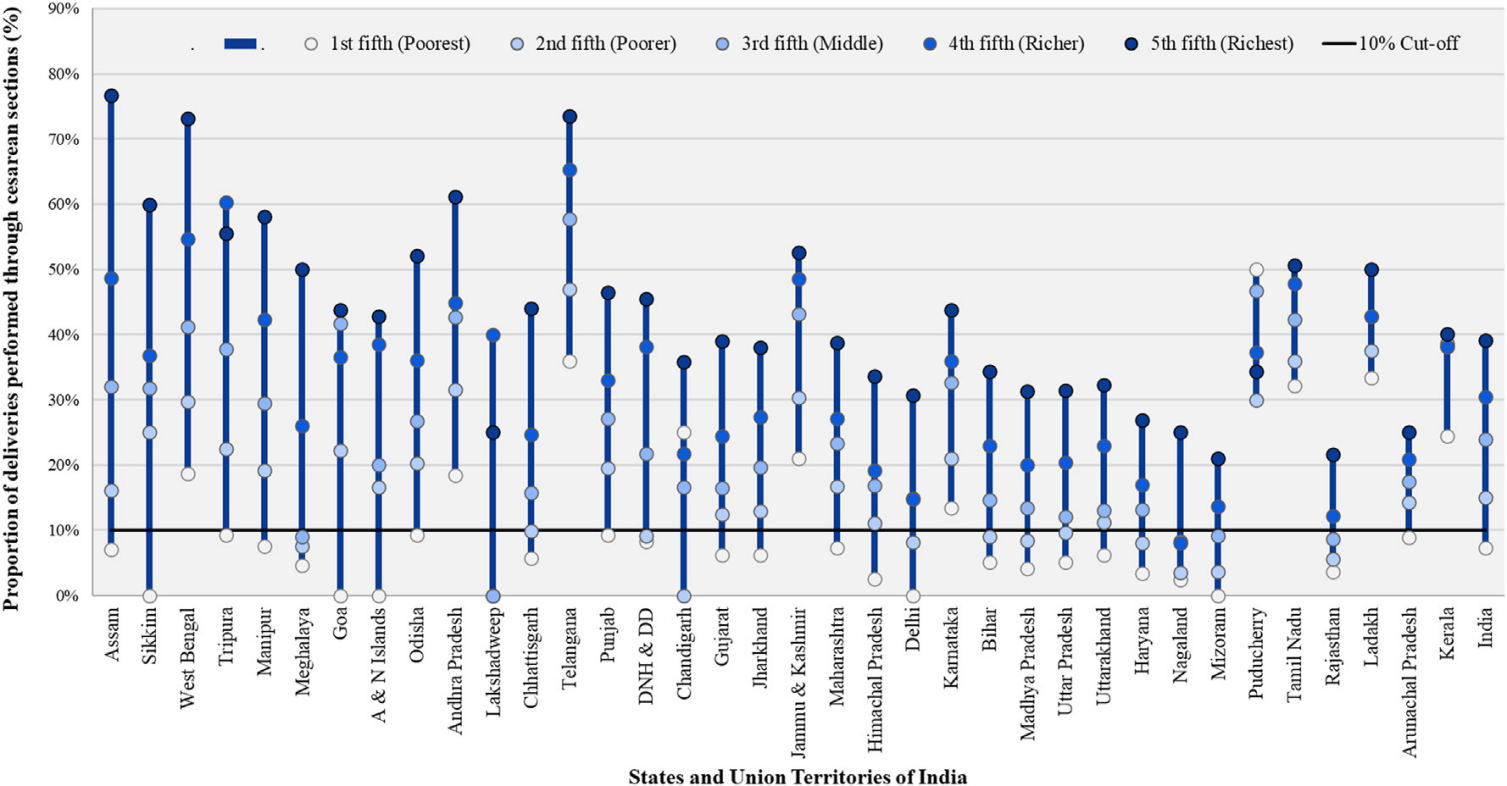
**Caesarean delivery rates by economic status across Indian states and union territories in decreasing order of inequality from 2019**–**2021.** The X-axis indicates states and UTs, with each state and UT represented by five circles (one for each wealth quintile group). Vertical blue lines indicate the difference between the minimum and maximum CD rates in each state and UT. The Y-axis indicates the percentage of deliveries performed through caesarean section, with each unit representing a 10% difference in the CD rate. States are arranged in decreasing order of the difference in CD rates between the richest and poorest wealth quintiles, highlighting the magnitude of disparity. A horizontal line at the 10% cut-off indicates the threshold for underuse of caesarean deliveries.

CD rates ranged from 0% in the poorest quintile to 76.7% in the richest quintile. Most of the states and UTs (69.4%) had CD rates that were at least twice as high in the richest fifth compared to the poorest fifth of the population (Fig. 2, Supplementary Table S1). In 75% of the states, the CD rate in the poorest wealth quintile was less than 10%. The national average rate for the poorest wealth quintile was five times lower than the richest [7.3% (95% CI: 7.1%–7.6%) compared with 39.1% (95% CI: 38.6%–39.6%)]. Assam had the largest difference in CD rates between the richest and poorest quintiles (69.7%) while Kerala had the least difference (15.7%) (Fig. 2, Supplementary Table S1).

### Relative inequalities in caesarean delivery rates

Fig. 1a and b shows the state-level variations of the CD rates and relative inequality in access to CD respectively. The average relative inequality across the country was found to be 5.3. The highest relative inequalities in CD rates were observed in Himachal Pradesh (13.4), Nagaland (10.4), Assam (10.9), and Meghalaya (10.9). Conversely, the southern states of Tamil Nadu (1.6), Kerala (1.6), and Telangana (2.0) exhibited the most equitable distribution.

**Fig. 3:**
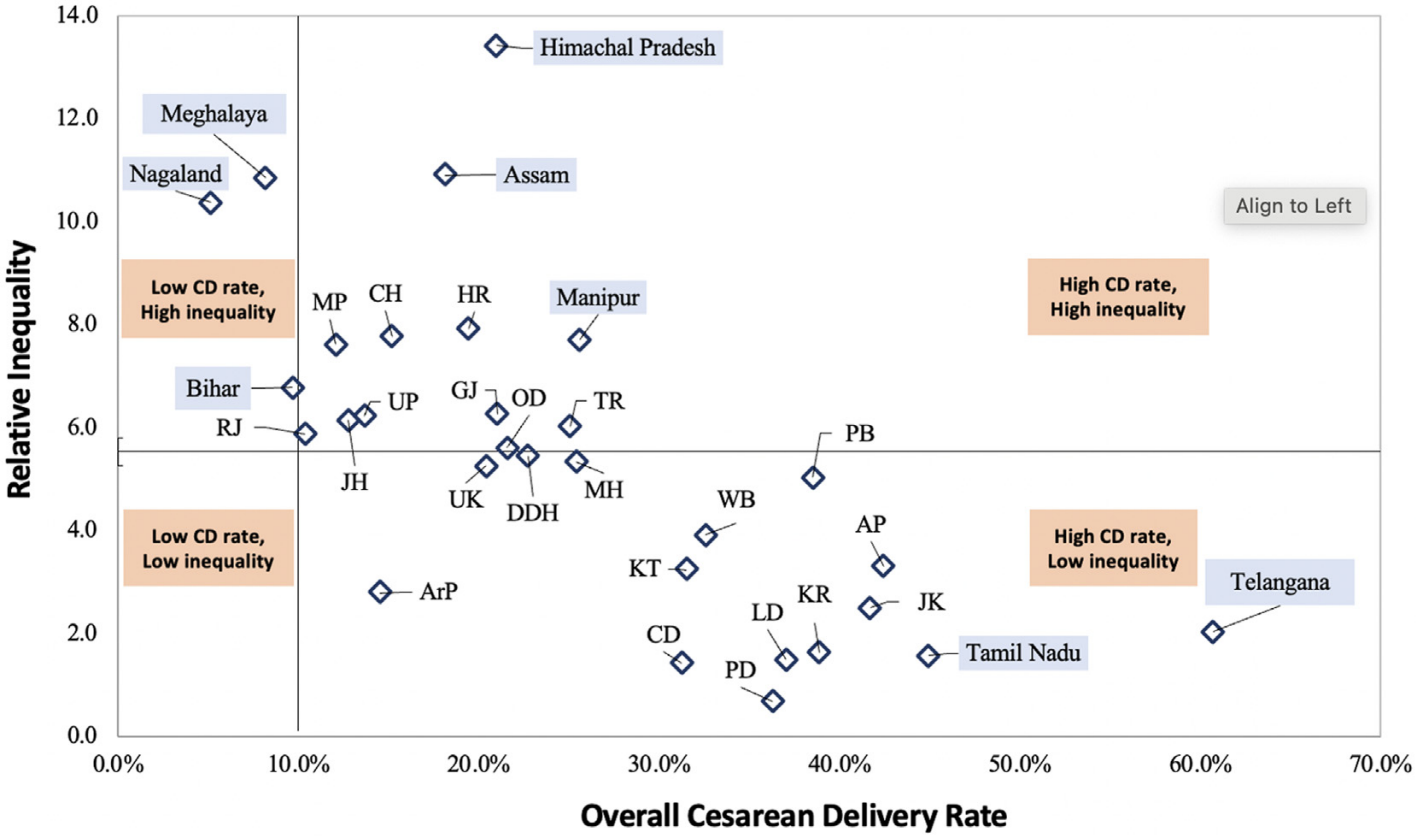
**Comparison of overall caesarean delivery rates (CD rates) and relative wealth-related inequality in caesarean deliveries across 36 states and union territories.** IN: India, AP: Andhra Pradesh, ArP: Arunachal Pradesh, AS: Assam, BI: Bihar, CH: Chhattisgarh, GJ: Gujarat, GO: Goa, HP: Himachal Pradesh, HR: Haryana, JH: Jharkhand, KL: Kerala, KT: Karnataka, ME: Meghalaya, MH: Maharashtra, MN: Manipur, MP: Madhya Pradesh, MZ: Mizoram, NG: Nagaland, OD: Odisha, PB: Punjab, RJ: Rajasthan, SK: Sikkim, TL: Telangana, TN: Tamil Nadu, TR: Tripura, UK: Uttarakhand, UP: Uttar Pradesh, WB: West Bengal, AN: Andaman & Nicobar Islands, CD: Chandigarh, DDH: Dadra Nagar Haveli & Diu Daman, DL: Delhi, JK: Jammu & Kashmir, LD: Ladakh, LK: Lakshadweep, PD: Puducherry. The vertical line passes through the 10% overall caesarean delivery rate and the horizontal line passes through the relative inequality median of 5.15. ∗The relative inequality in CD rates could not be calculated for six states and UTs: Sikkim, Goa, Andaman & Nicobar Islands, NCT of Delhi, Lakshadweep, and Mizoram, as the poorest had a CD rate of 0%. Here, 5.33 is the median relative inequality for all states and UTs. A caesarean delivery rate of 10% was chosen as the dividing point on the x-axis per WHO recommendations.

Fig. 3 depicts a scatter plot showing the states and UTs based on their CD rates and relative wealth-related inequality. We observed a clustering pattern of certain states with low CD rates exhibiting higher inequality (Nagaland, Meghalaya), high CD rates exhibiting high inequality (Himachal Pradesh, Assam, Manipur), and high CD rates exhibiting low inequality (Telangana, Tamil Nadu). The states with low relative inequalities (below the relative inequality median of 5.2) had CD rates ranging from 14.5% to 60.7%. In comparison, states with higher inequalities had CD rates in the range of 5.2%–25.6%.

### Caesarean delivery rates by type of healthcare facility

**Fig. 4:**
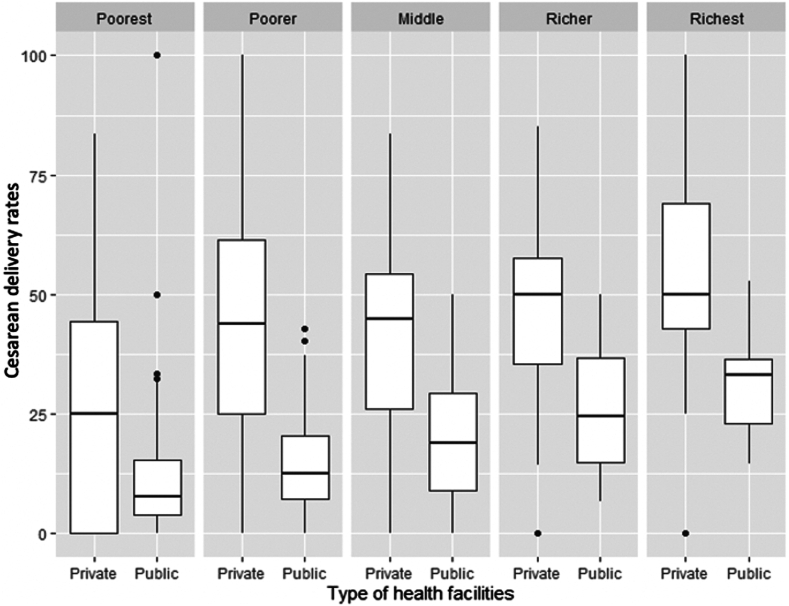
**A box-and-whisker plot for health sector-wise CD rates in all 36 States and Union Territories across wealth quintiles.** For each box-and-whisker plot, the horizontal bar indicates the median, the upper (third quartile) and lower limits (first quartile) of the box the interquartile range, and the ends of the whiskers from the bottom of the box to the top indicate the 5th percentile (minimum CD rate) and the 95th percentile (maximum CD rate). The black dots from the bottom of the box to the top represent the rates less than the 5th percentile or greater than the 95th percentile. Outliers, (represented as dots above or below the whiskers) are any value above or below Q3/Q1 ± 1.5 multiplied by IQR.

The average CD rate was 47.5% in private and 14.3% in public facilities. In both public and private facilities, CD rates increased from the poorest to the richest fifth quintiles of the population. The median CD rate was the highest for the richest quintile at 50% (IQR = 42.9%–69.1%) in private hospitals and 33% (IQR = 22.9%–36.6%) in public hospitals. The median CD rate in the poorest quintile was 25% (IQR = 0%–44.4%) in private hospitals and 7.7% (IQR = 4%–5.3%) in public hospitals (Fig. 4). At each wealth quintile, CD rates were higher in private compared to public facilities for 61.1% (22/36) of states and UTs.

The box plot revealed that from the poorest to richest quintile, the CD rates in public hospitals also increased. This rise is more pronounced compared to the relatively steady use of private hospitals for CDs across different wealth quintiles. The absolute number of births stratified by facility type and wealth quintile for all states and UTs are provided in Supplementary Table S2.

### Outliers

Only 5.6% of facilities in the private sector across India had CD rates below 10%. The CD rate in the private sector was 0% in Arunachal Pradesh and Ladakh. In Puducherry, with a CD rate of 36.3% and a relative inequality of 0.7, we observed a reverse trend where the CD rate in the poorest quintile (50%) was higher than in the richest quintile (34.9%) within public facilities, contrary to other states.

## Discussion

We found substantial geographical variations in CD rates across Indian states and UTs. Disaggregation of CD rates by wealth quintiles revealed inequality in rates, with the lowest rates in the poorest fifth and the highest in the richest fifth of the population in both public and private facilities. CD rates are highest in private facilities amongst the richest quintiles. Additionally, both people with high-income and people with low-income in India have higher CD rates in private facilities than in public facilities. This highlights the dual nature of healthcare in India, where private care is often seen as offering more immediate services, benefiting populations with high-income, while populations with low-income struggle to access timely and safe CDs.

As distinctive and nuanced as the state cultures, are the factors contributing to the disparity in CD rates across Indian states. Our findings reveal significant variations in CD rates across states, as seen in Fig. 1, Fig. 3. Despite the overall inverse correlation between CD rates and relative inequality, Arunachal Pradesh, a remote state comprising of indigenous population, shows a relatively lower CD rate of 14.5% and low relative inequality (2.81). A qualitative study in Arunachal Pradesh highlighted women’s preference for vaginal delivery due to perceived fear of long-term health issues, higher risks and costs associated with CDs.^31^ The low inequality in the state may result from challenging terrains limiting access to CD facilities uniformly across economic groups, though this is speculative and requires further investigation. In contrast, southern states such as Tamil Nadu and Telangana exhibit high CD rates and low inequality. This inverse correlation is potentially attributable to factors like improved healthcare accessibility, higher literacy, higher GDP, and provision of payments beyond the JSY scheme.^22^^,^^23^^,^^32^ Cultural and social factors such as fear of normal childbirth, the desire to deliver on an auspicious day, and a preference for painless deliveries and smaller families, also potentially contribute to higher CD rates.^33^ On the other hand, most of the population in Bihar, is engaged in daily wage work and is in the lower wealth quintiles.^34^^,^^35^ Therefore, a preference for vaginal deliveries due to their affordability and shorter recovery times, has been reported, even when CDs are medically recommended.^36^ North-eastern states like Nagaland with low CD rates and high inequality, face challenges tied to the *Rashtriya Swasthya Bima Yojana* (RSBY) insurance program. This is the primary health insurance scheme for those living below the poverty line in the region and has been found to negatively influence CD access as beneficiaries of the program were found to have lower CD rates than those not funded by the program.^37^^,^^38^ High financial constraints on patients, and a lack of awareness about available schemes often overshadow the benefits of insurance programs, like RSBY. As a result, beneficiaries turn to paid services instead of utilizing their insurance benefits. Insurance schemes like *Ayushman Bharat Pradhan Mantri Jan Arogya Yojana* (AB PM-JAY) enable economically disadvantaged groups to access private healthcare, but for-profit private hospital deliveries still result in higher out-of-pocket expenses, causing financial distress.^39^ The inverse correlation of low CD rates and high inequality may also result from the richest quintile being more likely to secure CD services in high-demand, low-supply situations, potentially exacerbating inequality. Conversely, higher CD rates with low inequality might indicate better capacity, which could reduce unmet needs and extend services to underserved populations, possibly lowering inequality. However, states with both high CD rates and high inequality suggest that this explanation may not fully account for all disparities.

We demonstrate that CD rates in the private sector are higher than in the public sector across all wealth quintiles in India. This mirrors trends noted in other research reaffirming that wealthier populations are linked to increased CD access.^3^^,^^23^^,^^40^ This finding is also supported by research from studies in low-income and middle-income countries such as Bangladesh, Brazil, Ghana, Nigeria, and Indonesia.^41^, ^42^, ^43^, ^44^ Additionally we found that the richest quintiles in private hospitals have higher CD rates when compared to public hospitals.

In public hospitals, CD rates are lowest in the poorest quintiles and increase progressively from the poorest to the richest quintiles. This trend may partly be explained by individuals with high-income having better access to insurance schemes or ease of referrals, leading to higher caesarean delivery rates. The high CD rates among the poorest in the private sector may stem from both patient and provider factors. While the referral of complicated pregnancies to cause this high rate has been countered,^45^ other patient-related reasons such as fear of normal deliveries or cultural preferences may exist and require further qualitative studies. On the provider side, personnel qualified to perform CDs are more likely to be employed in the private sector^46^ due to a lack of economic incentives for practitioners.^47^ Additionally, clinicians prefer CDs due to perceived time taken, doctor’s convenience, lack of context specific guidelines, socio-cultural reasons, and fears of litigation over potential adverse outcomes in normal deliveries leading to procedure overuse.^48^ This phenomenon is possibly influenced by rampant workplace violence experienced by doctors and health workers in Indian settings.^49^^,^^50^ Similar concerns about litigation influence delivery choices globally.^51^

Understanding the absolute number of births alongside CD rates, stratified by facility type and wealth quintile, offers more insights into accessibility. From our analysis, in the public sector, births decrease from the poorest to the richest quintile (41,825–13,975), while in the private sector, they increase (4158–15,865), highlighting the shift of reliance toward private facilities among populations with high-income. The poorest quintile predominantly relies on public healthcare facilities and is disproportionately impacted by the limited availability and/or subpar quality of care in public establishments. This disparity accentuates persistent gaps in the accessibility, quality, and utilization of public healthcare facilities for CDs. Additionally, seeking healthcare in private facilities incurs elevated costs. It is associated with financial distress which is notably higher at 27.0% in private health facilities compared to 16.6% in public facilities for CDs.^52^ Despite conditional cash incentives in public facilities, women still face financial distress paying up to half of their received incentive towards delivery care.^53^ The average overall out-of-pocket expenditure for CDs is eight times higher than for normal vaginal deliveries, posing a significant risk of impoverishment, especially for lower-income groups.^54^ This risk is positively correlated with higher birth orders, lower education, and lower socioeconomic status among women, intensifying disparities in access.^52^^,^^55^ Disparities in maternal healthcare access are intricately tied to economic status, gender, and caste, and overlooking these structural determinants perpetuates inequities in policy design and implementation.^56^ Denial of CD when clinically indicated reflects inequality, regardless of the facility type. This may result from systemic failures rather than solely provider’s prejudices.^12^ While several factors are responsible for the widening public-private CD disparity, the effectiveness of government programs that aim to counter financial barriers to providing CDs in underserved areas comes into question.^19^^,^^20^^,^^57^^,^^58^ At the same time, we must recognise that without knowing a ‘baseline effective CD rate’ for a facility, we cannot determine the true effectiveness of a policy. Several other factors exist and must be examined to understand the disparity between the rich and poor delivering at the same facility.

India is projected to have the highest number of CDs worldwide by 2030, with persisting disparities favouring populations of high socioeconomic status.^59^ States like Gujarat and Madhya Pradesh have introduced public-private partnerships to increase access to CD for poorer populations and marginalized communities. In Gujarat, an initiative was implemented to increase access to emergency obstetric care (including CDs).^60^ Under this scheme, the state pays for CDs in private hospitals for indigenous and below-poverty-line populations. However, there is some evidence to suggest that the program may not be meeting its intended goals of increasing CD rates in the target populations.^61^ For example, a study found that women not covered by the initiative had significantly higher odds of undergoing a CD compared to those who were covered.^62^ In addition, obstetricians did not have an incentive to provide a CD.^62^ Similarly, in Madhya Pradesh, higher reimbursements provided to patients under JSY may inadvertently be elevating CD rates contributing to the trend of overuse while still failing to reach the target population groups needing the procedure.^40^ Policy failures often stem from multiple factors, requiring further research using implementation evaluation and policy analysis frameworks. Periodically assessing the implementation and effectiveness of health policies can improve outcomes for patients, ensuring they align with their intended goals.

While no single prescriptive solution exists, evidence-based strategies in similar contexts can provide learning points to balance the under- and overuse of CDs. To curb unnecessary CDs, we recommend adopting and implementing WHO evidence-based non-clinical guidelines, including mandatory second opinions, periodic audits, and physician education.^63^^,^^64^ WHO statement on caesarean section rates *“Every effort should be made to provide caesarean sections to women in need, rather than striving to achieve a specific rate,”*^65^ underscores the importance of focusing on clinical need rather than any particular thresholds. In the Indian context, several interventions can be implemented to enhance clinicians’ understanding of CD decision-making: (i) recording second opinions for elective CDs, (ii) establishing transparent and detailed informed consent processes to reduce defensive medicine practices,^66^^,^^67^ and (iii) adopting a stringent electronic monitoring and reporting system for CD rates and outcomes at institutional, district, and state levels utilising the Robson classification.^65^^,^^68^ Utilising such comparative frameworks would allow for a more accurate assessment of policy outcomes, contributing to more equitable access to CD services. Furthermore, achieving transparency in obstetric outcome data, such as maternal and perinatal mortality rates, through non-punitive reviews is critical for comprehensive insights of their relationship with varying CD rates.^56^ The variations in CD rates described in this study should be interpreted considering socio-demographic,^22^^,^^23^ cultural, psychological, and behavioural factors. Future research using participatory action research, ethnographic studies, and qualitative interviews is recommended.

This study utilised data from India’s largest publicly available dataset focusing on the nationwide disparity in access to CDs using wealth quintile and health facility type (public and private). However, limitations include potential biases in NFHS-5 data due to sampling, non-response, social desirability, and recall biases. Mitigation measures within the survey include utilising a representative sample, anonymous questionnaires for sensitive queries, maximizing response rates, and cross-verification with official records. Standardized questionnaires and procedures used in the survey aim to enhance result accuracy and consistency. As a descriptive cross-sectional analysis, this study is limited in its ability to infer causality and cannot explain the entirety of the underlying causes of the observed CD rate disparities. We have utilised population-level CD rates and not facility-based reported CD rates which means it is not enumeration and is a representative sample. Relative inequality in CD rates could not be calculated for six states as the CD rates in those states are not available. Finally, in Puducherry, a small number of births in the poorest with a high CD rate (50%) may not be entirely representative of the broader population in this quintile and may have contributed to the reverse trend of higher utilisation of CDs by the poorest quintile in comparison to the richest quintile in this UT.

In conclusion, state-level CD rates in India mask disparities in access among the people with low-income as they are offset by high caesarean use amongst the people with high-income. The anomalous increase in CD rates alongside the continued disparities by wealth quintiles warrants future work to examine the influence of structural determinants in accessing CDs. At the national and state level, we recommend monitoring government schemes that provide or incentivise CDs to understand their influence on CD rates and access, with contextual state-level policy refinements. At the local and hospital levels, we recommend the implementation of the Robson Classification to gain insights into clinicians and hospital-level influence on CD rates.

## Contributors

RD, PN, PP, and AG conceptualised the study. RD, PN, and PP analysed the data with support from AG, RG, ST, NR, JB, AAB, and AvD and verified underlying data. All co-authors provided input to the first draft, reviewed successive drafts, and made revisions. All authors approved the manuscript for submission. RD and PN contributed equally and are co-first authors. AG and AAB also contributed equally and are co-senior authors.

## Data sharing statement

This study used publicly available data from the National Family Health Survey 2019–2021, which can be accessed via the Demographic and Health Surveys (DHS) Program at https://dhsprogram.com/. The analysis code is publicly available on GitHub: https://github.com/GlobalSurgery/NFHS-5-State-wise-variation-and-inequalities-in-caesarean-delivery-rates-in-India and also included in the Supplementary information.

## Editor note

The Lancet Group takes a neutral position with respect to territorial claims in published maps and institutional affiliations.

## Declaration of interests

The authors declare no conflicts of interest.
