# Supplementary material for: State-wise variation and inequalities in caesarean delivery rates in India: analysis of the National Family Health Survey-5 (2019–2021) data
Source: Lancet Reg Health Southeast Asia. 2024 Dec 3;32:100512. doi: 10.1016/j.lansea.2024.100512 (PMC11665369; doi:10.1016/j.lansea.2024.100512)
Supplement: Supplementary Table S1 [file mmc1.docx]

**Supplementary Table 1: Overall caesarian delivery (CD) rates and wealth inequalities in CD rates across states and UTs in India arranged in descending order of relative inequality**

**denotes union territories (UTs). Note: % are based on weighted samples.* *These CD rates represent the percentage of cesarean deliveries among total births in the respective wealth quintiles or health sectors and should not be interpreted as the distribution of cesarean deliveries across these facilities. Relative inequalities were unavailable for Sikkim, Goa, Andaman & Nicobar Islands, National Capital Territory of Delhi (NCT of Delhi), Lakshadweep, and Mizoram.*

| **State** | **Poorest** |  | **Poorer** |  | **Middle** |  | **Richer** |  | **Richest** |  | **Total** |  | (Richest / Poorest) |
| --- | --- | --- | --- | --- | --- | --- | --- | --- | --- | --- | --- | --- | --- |
|  | **% (95% CI)** | **# Births** | **% (95% CI)** | **# Births** | **% (95% CI)** | **# Births** | **% (95% CI)** | **# Births** | **% (95% CI)** | **# Births** | **% (95% CI)** | **# Births** | **Relative Diff** |
| Andhra Pradesh | 18.5 (12 - 24.9) | 139 | 31.5 (27.8 - 35.3) | 592 | 42.7 (39.5 - 45.8) | 946 | 44.9 (41.5 - 48.4) | 787 | 61.2 (56.2 - 66.1) | 369 | 42.4 (40.6 - 44.3) | 2833 | 3.31 |
| Arunachal Pradesh | 8.9 (7.4 - 10.4) | 1384 | 14.3 (12.7 - 15.9) | 1770 | 17.5 (15.5 - 19.5) | 1324 | 20.8 (18.1 - 23.6) | 837 | 25 (19.1 - 30.9) | 209 | 14.5 (13.6 - 15.5) | 5524 | 2.81 |
| Assam | 7 (6.3 - 7.7) | 4842 | 16.1 (14.9 - 17.3) | 3437 | 32 (29.6 - 34.4) | 1447 | 48.7 (45 - 52.4) | 698 | 76.7 (71.1 - 82.3) | 221 | 18.1 (17.4 - 18.9) | 10645 | 10.92 |
| Bihar | 5.1 (4.6 - 5.5) | 10530 | 9.1 (8.3 - 9.8) | 5683 | 14.7 (13.4 - 16) | 2758 | 23 (20.9 - 25.1) | 1525 | 34.4 (30.4 - 38.4) | 544 | 9.7 (9.3 - 10.1) | 21040 | 6.78 |
| Chhattisgarh | 5.7 (4.9 - 6.4) | 3532 | 9.9 (8.6 - 11.2) | 1968 | 15.7 (13.8 - 17.7) | 1389 | 24.7 (22.1 - 27.3) | 1029 | 44.1 (40.1 - 48.1) | 596 | 15.2 (14.4 - 16) | 8514 | 7.78 |
| Goa | 0 (0 - 0) | 7 | 22.2 (3 - 41.4) | 18 | 41.7 (26.9 - 56.4) | 43 | 36.5 (26.6 - 46.5) | 90 | 43.8 (37.1 - 50.4) | 211 | 39.3 (34.3 - 44.3) | 369 | - |
| Gujarat | 6.2 (5.1 - 7.3) | 1813 | 12.5 (11.1 - 13.9) | 2200 | 16.5 (14.9 - 18.1) | 2144 | 24.5 (22.6 - 26.3) | 2073 | 39 (36.6 - 41.3) | 1638 | 21 (20.2 - 21.8) | 9868 | 6.27 |
| Haryana | 3.4 (0.9 - 5.9) | 206 | 8.1 (6.1 - 10.1) | 713 | 13.2 (11.3 - 15.1) | 1167 | 16.9 (15.2 - 18.6) | 1904 | 26.9 (25.3 - 28.5) | 2925 | 19.4 (18.5 - 20.4) | 6915 | 7.93 |
| Himachal Pradesh | 2.5 (0.1 - 4.9) | 167 | 11.1 (8.3 - 13.9) | 472 | 16.9 (14 - 19.8) | 635 | 19.2 (16.4 - 22.1) | 738 | 33.6 (29.9 - 37.3) | 623 | 21 (19.4 - 22.5) | 2635 | 13.43 |
| Jammu & Kashmir | 21 (18.3 - 23.7) | 886 | 30.3 (27.7 - 32.9) | 1201 | 43.2 (40.4 - 45.9) | 1233 | 48.6 (46 - 51.2) | 1430 | 52.5 (49.6 - 55.5) | 1107 | 41.7 (40.4 - 43) | 5857 | 2.50 |
| Jharkhand | 6.2 (5.5 - 6.8) | 5523 | 13 (11.6 - 14.4) | 2221 | 19.7 (17.5 - 22) | 1213 | 27.4 (24.1 - 30.6) | 733 | 38 (32.9 - 43) | 357 | 12.8 (12.1 - 13.4) | 10047 | 6.15 |
| Karnataka | 13.4 (11 - 15.9) | 749 | 21 (19.1 - 22.8) | 1863 | 32.7 (30.9 - 34.5) | 2642 | 36 (34 - 38) | 2146 | 43.7 (40.6 - 46.8) | 983 | 31.5 (30.5 - 32.5) | 8383 | 3.26 |
| Kerala | 24.4 (10.8 - 38.1) | 38 | 38.1 (29 - 47.3) | 108 | 38.6 (34.2 - 43.1) | 462 | 38.1 (35.2 - 41.1) | 1061 | 40.1 (37.2 - 43.1) | 1065 | 38.9 (37.1 - 40.7) | 2734 | 1.64 |
| Madhya Pradesh | 4.1 (3.6 - 4.6) | 6153 | 8.4 (7.6 - 9.3) | 3875 | 13.4 (12 - 14.7) | 2582 | 20 (18.3 - 21.7) | 2116 | 31.3 (29 - 33.6) | 1554 | 12.1 (11.6 - 12.6) | 16280 | 7.62 |
| Maharashtra | 7.3 (5.8 - 8.8) | 1144 | 16.8 (15.1 - 18.4) | 1921 | 23.3 (21.6 - 25) | 2446 | 27.1 (25.4 - 28.9) | 2490 | 38.8 (36.4 - 41.3) | 1519 | 25.4 (24.5 - 26.3) | 9520 | 5.34 |
| Manipur | 7.5 (5.9 - 9.2) | 1001 | 19.2 (16.8 - 21.6) | 1060 | 29.4 (26 - 32.9) | 667 | 42.3 (37.1 - 47.4) | 358 | 58.1 (49.9 - 66.3) | 139 | 25.6 (24.1 - 27.1) | 3225 | 7.71 |
| Meghalaya | 4.6 (3.9 - 5.3) | 3108 | 7.5 (6.5 - 8.6) | 2285 | 9 (7.1 - 10.9) | 895 | 26.1 (21 - 31.2) | 284 | 50 (36.9 - 63.1) | 56 | 8.2 (7.5 - 8.8) | 6628 | 10.86 |
| Mizoram | 0 (0 - 0) | 335 | 3.6 (2 - 5.2) | 507 | 9.1 (6.9 - 11.2) | 690 | 13.7 (11 - 16.4) | 618 | 21.1 (16.5 - 25.6) | 304 | 11.1 (9.9 - 12.4) | 2454 | - |
| Nagaland | 2.4 (1.6 - 3.2) | 1412 | 3.5 (2.3 - 4.7) | 875 | 8.3 (5.7 - 11) | 426 | 8 (4.6 - 11.4) | 247 | 25 (16.2 - 33.8) | 92 | 5.2 (4.4 - 5.9) | 3052 | 10.38 |
| Odisha | 9.3 (8.3 - 10.2) | 3514 | 20.3 (18.6 - 22) | 2145 | 26.8 (24.5 - 29.1) | 1438 | 36.1 (33 - 39.1) | 948 | 52.1 (47.6 - 56.6) | 477 | 21.6 (20.7 - 22.5) | 8522 | 5.61 |
| Punjab | 9.2 (3.5 - 15) | 98 | 19.5 (15.6 - 23.4) | 394 | 27.1 (23.9 - 30.3) | 746 | 33 (30.5 - 35.5) | 1347 | 46.5 (44.7 - 48.2) | 3031 | 38.5 (37.3 - 39.8) | 5616 | 5.03 |
| Rajasthan | 3.7 (3 - 4.4) | 2603 | 5.6 (4.9 - 6.4) | 3444 | 8.7 (7.7 - 9.6) | 3293 | 12.1 (11 - 13.3) | 2971 | 21.6 (20 - 23.3) | 2332 | 10.4 (9.9 - 10.9) | 14643 | 5.88 |
| Sikkim | 0 (0 - 0) | 39 | 25 (18.4 - 31.6) | 166 | 31.8 (26.1 - 37.5) | 256 | 36.8 (28.6 - 45.1) | 130 | 60 (42.2 - 77.8) | 29 | 32.8 (29.1 - 36.5) | 620 | - |
| Tamil Nadu | 32.1 (25.9 - 38.4) | 214 | 35.9 (32.9 - 39) | 942 | 42.3 (40.1 - 44.5) | 1985 | 47.8 (45.7 - 50) | 2090 | 50.7 (47.9 - 53.4) | 1267 | 44.9 (43.7 - 46.1) | 6498 | 1.58 |
| Telangana | 36 (31.3 - 40.7) | 398 | 46.9 (44.3 - 49.6) | 1375 | 57.7 (55.7 - 59.7) | 2337 | 65.3 (63.3 - 67.3) | 2127 | 73.6 (71 - 76.2) | 1081 | 60.7 (59.6 - 61.8) | 7318 | 2.04 |
| Tripura | 9.2 (7.3 - 11.2) | 835 | 22.5 (19.3 - 25.7) | 665 | 37.8 (33 - 42.5) | 401 | 60.3 (52.6 - 68.1) | 153 | 55.6 (33.8 - 77.3) | 20 | 25.1 (23.2 - 26.9) | 2074 | 6.03 |
| Uttar Pradesh | 0 (0 - 0) | 10049 | 0 (0 - 0) | 9444 | 12.1 (11.3 - 12.8) | 6561 | 20.4 (19.3 - 21.5) | 5208 | 31.4 (30.1 - 32.8) | 4504 | 13.7 (13.3 - 14) | 35766 | 6.23 |
| Uttarakhand | 6.1 (3.4 - 8.9) | 285 | 11.2 (9.2 - 13.2) | 933 | 13 (11 - 15.1) | 1028 | 23 (20 - 26) | 760 | 32.3 (29 - 35.6) | 778 | 20.4 (19.1 - 21.7) | 3784 | 5.26 |
| West Bengal | 18.7 (17 - 20.3) | 2191 | 29.7 (27.5 - 32) | 1625 | 41.2 (38.1 - 44.2) | 999 | 54.7 (50.6 - 58.8) | 567 | 73.2 (67.6 - 78.9) | 236 | 32.6 (31.4 - 33.8) | 5618 | 3.92 |
| Andaman & Nicobar Islands | 0 (0 - 0) | 60 | 16.7 (9.3 - 24) | 98 | 20 (13.1 - 26.9) | 129 | 38.5 (29.7 - 47.2) | 118 | 42.9 (29.9 - 55.8) | 56 | 30 (25.8 - 34.2) | 461 | - |
| Chandigarh | 25 (-13 - 63) | 5 | 0 (0 - 0) | 3 | 16.7 (-6.4 - 39.8) | 10 | 21.7 (6.2 - 37.3) | 27 | 35.8 (27.5 - 44.1) | 129 | 31.3 (24.4 - 38.1) | 174 | 1.43 |
| Dadra & Nagar Haveli And Daman & Diu | 8.3 (1.4 - 15.3) | 61 | 9.1 (4.4 - 13.8) | 144 | 21.7 (15.8 - 27.6) | 188 | 38.1 (31.9 - 44.3) | 236 | 45.5 (37.9 - 53) | 166 | 22.7 (19.8 - 25.6) | 795 | 5.45 |
| Delhi | 0 (0 - 0) | 10 | 8.2 (3.3 - 13.1) | 120 | 14.9 (11.2 - 18.6) | 357 | 14.8 (12.2 - 17.4) | 716 | 30.7 (28.6 - 32.9) | 1734 | 23.6 (22.1 - 25.1) | 2937 | - |
| Ladakh | 33.3 (21.8 - 44.9) | 64 | 37.5 (29.7 - 45.3) | 147 | 42.9 (34.5 - 51.2) | 134 | 42.9 (34.7 - 51.1) | 140 | 50 (35.2 - 64.8) | 44 | 37 (32.9 - 41.2) | 529 | 1.50 |
| Lakshadweep | 0 (0 - 0) | 0 | 0 (0 - 0) | 4 | 0 (0 - 0) | 37 | 40 (31.4 - 48.6) | 124 | 25 (16.9 - 33.1) | 111 | 30 (24.6 - 35.4) | 276 | - |
| Puducherry | 50 (20.5 - 79.5) | 11 | 30 (16.6 - 43.4) | 45 | 46.7 (35.4 - 58) | 75 | 37.3 (31.5 - 43) | 268 | 34.3 (29.5 - 39.2) | 367 | 36.3 (32.9 - 39.7) | 766 | 0.69 |
| India | 7.3 (7.1 - 7.5) | 63406 | 15 (14.7 - 15.3) | 54463 | 23.9 (23.5 - 24.3) | 45083 | 30.4 (29.9 - 30.9) | 39094 | 39.1 (38.6 - 39.7) | 30874 | 21.5 (21.3 - 21.7) | 232920 | 5.33 |
