# Supplementary material for: State-wise variation and inequalities in caesarean delivery rates in India: analysis of the National Family Health Survey-5 (2019–2021) data
Source: Lancet Reg Health Southeast Asia. 2024 Dec 3;32:100512. doi: 10.1016/j.lansea.2024.100512 (PMC11665369; doi:10.1016/j.lansea.2024.100512)
Supplement: Supplementary Table S2 [file mmc2.docx]

**Supplementary Table S2: Proportion of total institutional live births by Cesarean Delivery (CD) in the respective Wealth Quintiles and in type of healthcare sectors in 36 Indian States and UTs** **denotes union territories (UTs). % are based on the weighted samples. These CD rates represent the percentage of C-sections among total births in the respective wealth quintiles or health sectors and should not be interpreted as the distribution of C-sections across these facilities.*

|  | **Poorest** |  |  |  |  | **Poorer** |  |  |  | **Middle** |  |  |  | **Richer** |  |  |  | **Richest** |  |  |  |
| --- | --- | --- | --- | --- | --- | --- | --- | --- | --- | --- | --- | --- | --- | --- | --- | --- | --- | --- | --- | --- | --- |
|  | **Public** |  |  | **Private** |  | **Public** |  | **Private** |  | **Public** |  | **Private** |  | **Public** |  | **Private** |  | **Public** |  | **Private** |  |
|  | **%** |  | **# Births** | **%** | **# Births** | **%** | **# Births** | **%** | **# Births** | **%** | **# Births** | **%** | **# Births** | **%** | **# Births** | **%** | **# Births** | **%** | **# Births** | **%** | **# Births** |
| Andhra Pradesh | 15.99 |  | 99 | 65.85 | 16 | 22.24 | 399 | 64.09 | 150 | 28.92 | 508 | 64.12 | 399 | 27.25 | 333 | 59.93 | 441 | 40.59 | 82 | 69.12 | 279 |
| Arunachal Pradesh | 15.38 |  | 798 | 0.00 | 12 | 16.28 | 1380 | 50.00 | 35 | 17.65 | 1130 | 50.00 | 53 | 20.00 | 707 | 50.00 | 84 | 16.67 | 162 | 0.00 | 46 |
| Assam | 8.37 |  | 3536 | 46.15 | 101 | 15.03 | 2879 | 62.50 | 186 | 25.12 | 1100 | 72.14 | 263 | 37.74 | 417 | 72.08 | 253 | 47.22 | 59 | 86.36 | 158 |
| Bihar | 2.56 |  | 6343 | 36.47 | 1020 | 3.67 | 3596 | 37.98 | 999 | 5.26 | 1561 | 40.03 | 800 | 6.73 | 691 | 43.57 | 668 | 14.59 | 161 | 43.09 | 347 |
| Chhattisgarh | 4.88 |  | 2488 | 53.85 | 121 | 6.86 | 1532 | 49.15 | 174 | 9.10 | 1044 | 52.44 | 219 | 14.90 | 747 | 57.30 | 234 | 23.56 | 286 | 64.09 | 291 |
| Goa | 0.00 |  | 7 | 0.00 | 0 | 12.50 | 16 | 50.00 | 2 | 42.11 | 33 | 40.00 | 10 | 25.71 | 67 | 52.94 | 23 | 36.17 | 91 | 49.23 | 119 |
| Gujarat | 3.97 |  | 1057 | 15.53 | 399 | 8.96 | 1220 | 18.17 | 766 | 9.78 | 1032 | 25.39 | 939 | 18.67 | 784 | 28.92 | 1196 | 26.39 | 364 | 43.66 | 1199 |
| Haryana | 5.06 |  | 133 | 10.00 | 15 | 8.16 | 470 | 16.67 | 110 | 8.08 | 824 | 35.06 | 237 | 10.35 | 1258 | 32.36 | 573 | 16.94 | 1342 | 35.72 | 1532 |
| Himachal Pradesh | 4.76 |  | 102 | 0.00 | 1 | 11.46 | 342 | 57.14 | 20 | 15.79 | 485 | 52.38 | 58 | 16.37 | 557 | 46.34 | 125 | 25.43 | 389 | 54.02 | 207 |
| Jammu & Kashmir | 27.04 |  | 664 | 50.00 | 8 | 33.20 | 1010 | 100.00 | 19 | 45.45 | 1116 | 77.78 | 34 | 48.43 | 1306 | 73.91 | 79 | 48.50 | 942 | 88.24 | 143 |
| Jharkhand | 4.99 |  | 3301 | 44.84 | 356 | 6.83 | 1423 | 43.83 | 395 | 10.20 | 736 | 49.00 | 328 | 9.31 | 360 | 50.94 | 316 | 23.71 | 118 | 46.31 | 221 |
| Karnataka | 12.62 |  | 613 | 29.21 | 72 | 17.64 | 1435 | 40.93 | 311 | 26.51 | 1936 | 54.22 | 628 | 25.08 | 1260 | 52.72 | 855 | 24.30 | 398 | 57.64 | 569 |
| Kerala | 19.44 |  | 32 | 44.44 | 6 | 40.21 | 80 | 34.15 | 28 | 38.58 | 247 | 38.62 | 212 | 37.96 | 400 | 38.66 | 652 | 35.17 | 202 | 41.46 | 854 |
| Madhya Pradesh | 4.21 |  | 4934 | 41.18 | 110 | 7.41 | 3374 | 42.66 | 177 | 9.47 | 2267 | 57.14 | 209 | 12.32 | 1687 | 52.52 | 373 | 17.77 | 950 | 54.34 | 551 |
| Maharashtra | 6.55 |  | 816 | 28.03 | 89 | 13.76 | 1425 | 34.00 | 348 | 20.00 | 1648 | 32.66 | 659 | 21.17 | 1411 | 35.51 | 1012 | 25.85 | 530 | 45.91 | 951 |
| Manipur | 14.63 |  | 354 | 20.00 | 69 | 20.37 | 656 | 50.00 | 101 | 27.94 | 380 | 50.00 | 167 | 36.84 | 178 | 53.33 | 148 | 46.15 | 51 | 66.67 | 81 |
| Meghalaya | 8.64 |  | 1198 | 33.33 | 93 | 8.44 | 1204 | 40.91 | 147 | 7.50 | 561 | 29.41 | 116 | 20.00 | 168 | 37.50 | 78 | 20.00 | 22 | 77.78 | 31 |
| Mizoram | 0.00 |  | 123 | 0.00 | 2 | 5.26 | 333 | 0.00 | 10 | 7.89 | 586 | 0.00 | 22 | 11.36 | 520 | 33.33 | 66 | 16.00 | 219 | 25.00 | 72 |
| Nagaland | 8.70 |  | 371 | 0.00 | 10 | 9.52 | 321 | 0.00 | 22 | 5.26 | 216 | 25.00 | 43 | 9.09 | 109 | 14.29 | 62 | 33.33 | 25 | 28.57 | 47 |
| Odisha | 8.47 |  | 2820 | 74.12 | 99 | 15.67 | 1856 | 72.39 | 181 | 18.80 | 1172 | 66.67 | 236 | 24.39 | 695 | 71.74 | 239 | 33.07 | 236 | 71.04 | 236 |
| Punjab | 7.69 |  | 61 | 25.00 | 20 | 16.47 | 269 | 44.00 | 72 | 27.58 | 549 | 38.95 | 134 | 27.07 | 897 | 57.63 | 353 | 35.66 | 1353 | 57.19 | 1615 |
| Rajasthan | 3.19 |  | 2184 | 12.23 | 197 | 4.99 | 2906 | 15.03 | 313 | 5.96 | 2666 | 25.96 | 462 | 8.18 | 2268 | 26.99 | 607 | 15.55 | 1542 | 34.87 | 743 |
| Sikkim | 0.00 |  | 33 | 0.00 | 3 | 20.00 | 150 | 100.00 | 10 | 29.41 | 217 | 50.00 | 28 | 37.50 | 110 | 50.00 | 19 | 33.33 | 15 | 100.00 | 11 |
| Tamil Nadu | 31.73 |  | 193 | 61.11 | 13 | 31.93 | 829 | 73.29 | 103 | 37.43 | 1545 | 61.65 | 432 | 36.95 | 1321 | 67.90 | 764 | 36.59 | 469 | 60.94 | 791 |
| Telangana | 32.45 |  | 267 | 74.42 | 75 | 37.22 | 892 | 75.95 | 415 | 43.91 | 1298 | 81.80 | 959 | 49.78 | 907 | 80.67 | 1152 | 52.73 | 283 | 84.32 | 776 |
| Tripura | 10.43 |  | 611 | 37.50 | 36 | 21.21 | 556 | 61.54 | 55 | 32.35 | 322 | 73.68 | 63 | 50.00 | 96 | 85.00 | 53 | 50.00 | 12 | 100.00 | 7 |
| Uttar Pradesh | 2.88 |  | 6589 | 28.66 | 1028 | 4.69 | 6156 | 34.90 | 1671 | 5.96 | 4074 | 35.36 | 1481 | 10.40 | 2826 | 43.01 | 1709 | 15.21 | 1710 | 45.46 | 2448 |
| Uttarakhand | 7.58 |  | 177 | 16.67 | 22 | 11.05 | 576 | 47.06 | 70 | 7.44 | 696 | 45.00 | 132 | 15.70 | 498 | 43.57 | 191 | 22.95 | 391 | 42.91 | 353 |
| West Bengal | 15.28 |  | 1730 | 83.63 | 161 | 23.13 | 1260 | 80.38 | 226 | 29.37 | 720 | 83.47 | 235 | 37.16 | 319 | 82.19 | 236 | 48.94 | 71 | 87.08 | 158 |
| A & N Islands | 0.00 |  | 56 | 0.00 | 0 | 20.00 | 97 | 0.00 | 0 | 20.00 | 128 | 0.00 | 0 | 36.36 | 102 | 66.67 | 16 | 25.00 | 36 | 100.00 | 19 |
| Chandigarh | 100.00 |  | 1 | 0.00 | 0 | 0.00 | 2 | 0.00 | 0 | 16.67 | 9 | 0.00 | 0 | 21.05 | 22 | 25.00 | 5 | 33.70 | 108 | 50.00 | 21 |
| DNH And DD | 9.09 |  | 56 | 0.00 | 2 | 5.56 | 110 | 50.00 | 22 | 22.22 | 137 | 25.00 | 42 | 25.00 | 141 | 50.00 | 86 | 40.00 | 93 | 40.00 | 72 |
| Delhi | 0.00 |  | 5 | 0.00 | 1 | 7.32 | 71 | 25.00 | 18 | 11.61 | 246 | 48.21 | 51 | 14.83 | 518 | 24.11 | 105 | 21.85 | 987 | 46.40 | 675 |
| Ladakh | 33.33 |  | 63 | 0.00 | 0 | 42.86 | 141 | 0.00 | 0 | 42.86 | 124 | 0.00 | 0 | 42.86 | 131 | 0.00 | 1 | 50.00 | 42 | 0.00 | 1 |
| Lakshadweep | 0.00 |  | 0 | 0.00 | 0 | 0.00 | 4 | 0.00 | 0 | 0.00 | 27 | 0.00 | 10 | 33.33 | 91 | 0.00 | 32 | 33.33 | 57 | 50.00 | 54 |
| Puducherry | 50.00 |  | 10 | 0.00 | 1 | 22.22 | 36 | 100.00 | 9 | 50.00 | 62 | 0.00 | 13 | 33.33 | 189 | 55.56 | 78 | 34.09 | 177 | 34.78 | 187 |
| **India** | **6.11** |  | **41825** | **37.84** | **4158** | **11.41** | **39006** | **42.29** | **7165** | **17.74** | **31402** | **47.53** | **9674** | **20.91** | **24091** | **48.48** | **12884** | **24.87** | **13975** | **50.94** | **15865** |
